# Medicine

**Published:** 1896-09

**Authors:** R. Shingleton Smith


### Progress of the Medical Sciences.

---

### MEDICINE.

**The mechanico-physiological method of treatment of circulatory derangements** instituted by Professor M. J. Oertel, of Munich, described fully in Von Ziemssen's *Handbook of General Therapeutics* (vol. vii.), has long been known, and the results are well summarised in an elaborate table (page 257). By these methods it has been attempted for the first time by one of the most arduous kinds of exercises—viz., mountain climbing—to act immediately upon the heart-muscle, and thus to cause powerful contractions. These exercises have been found to accomplish everything expected from them, and have influenced not only the heart itself, but also the other parts and functions of the body which influence the circulation—to wit, the expansion of the thorax, the enlargement of the respiratory surface by the forced inspiration, the greater fulness of the arterial system, the greater supply of oxygen, and the increased oxidation.

"The influence of mountain ascents of one thousand metres or more above the valley level upon the heart and lungs is a more powerful one than we can obtain by any other means. Such a perfect readjustment of circulatory derangement of so high a grade as described in the above cases has never hitherto been effected, and it well shows what great modifications of the organism and what radical reconstructions are possible by physiological means. Dehydration and mountain climbing must henceforward be regarded as one chief aid in circulatory disease, venous stasis, cardiac debility (in tuberculosis), compression of the pulmonary circulation, diminished oxidation of the tissues, and fatty heart."

The principle embodied in the above quotation is at the foundation of the more modern system associated with the names of Nauheim and the brothers Schott, whose methods have of late attracted much attention. The success obtained by the Zander movement cure induced Dr. August Schott to devise a system of mild gymnastics or drill for the treatment of neurasthenic and hysterical patients, when it was found that the method originally devised for steadying and strengthening the nervous system proved to be a valuable adjunct to the bath treatment of cardiac disease. The employment of **regulated muscular exercises in the treatment of heart-disease** has now become an established method, and has received the enthusiastic support of the many physicians who have visited Nauheim to learn it. There appears to be no limit to the possibilities of improvement in cases of failing heart. Oertel's method was applicable only in those cases which were capable of more or less walking or climbing; but the Schotts' method has extended the range of treatment by exercises and baths to those patients who are too ill to take other kinds of exercise, and in whom failure of compensation has led to stagnation in various organs giving the usual phenomena present in advanced cases of heart-disease with failure of heart-power and its results.

The most recent evidence on this subject is that given in the discussion on the **diagnosis and treatment of heart failure**, which was opened by Professor Sir Thomas Grainger Stewart at the meeting of the British Medical Association in Carlisle. After an interesting, clear, and concise summary of the whole subject in which he emphasised the value of rest, more especially suitable to our hospital cases accustomed to hard laborious work, he proceeded to summarise the present views on exercises of all kinds, including—

1. Passive exercises and massage.
2. Movements with regulated resistance (Schott).
3. More active movements, as in hill climbing (Oertel).

The methods by which the effects of treatment were to be gauged were analysed, and it was Sir Grainger Stewart's belief that the alterations which the area of dulness showed under treatment were often quite unmistakable, and could not be explained in any other way except by changes in the heart itself. He had fully satisfied himself by observation on the dead body that he could rely upon percussion for determining the size and outline of the heart; but he also pointed out that Dr. Macintyre, of Glasgow, had been so successful in obtaining outlines of the viscera by Röntgen's rays, that we well might look to this method for ocular demonstration of variations in the heart's area. His experience of **the Schott method** had satisfied him (1) that in a large proportion of cases it effects immediate improvement of the heart, as shown by auscultation and percussion; (2) that in many cases the rhythm of the pulse improves and the heart becomes more vigorous; (3) that while the immediate effect is in a sense temporary, the heart rarely goes back to its previous condition of dilatation, but remains somewhat smaller than it had been before the exercises; and (4) that gradually improvement of a lasting kind sets in, so that the heart recovers its tone and the area of dulness diminishes. He concluded that the system of resisted movements appeared to be best suited to cases in which a certain power of heart exists, whilst the more passive exercises of massage would be applicable to weaker conditions of the organ.

A very important part of the treatment at **Nauheim** consists of **baths**, and the speaker was able to say that this method produced changes as definite in the area of cardiac dulness as those resulting from exercises. Artificial baths, salines with free carbonic acid, gave results in Edinburgh as successful as at

Nauheim, and these results, both from exercises and baths, either alone or in combination, might be looked for with absolute certainty.

Dr. Herringham [1] gave an interesting comparison between the ordinary percussion, which he found to be extraordinarily accurate, and that of auscultatory percussion, which was found to have many difficulties and drawbacks. On this question there is much diversity of opinion and practice, and Dr. Bezly Thorne's [2] views thereon are now well known. The demonstration given by Dr. Thorne [3] at the Cumberland Infirmary must have convinced the incredulous that both baths and exercises are potent instruments in producing a profound modification in the circulation as shown both by pulse tracings and by Dr. Thorne's method of mapping out the heart by means of auscultatory percussion. It is an astonishing fact that a few simple exercises (seven of which were performed in the case demonstrated) should produce so much result. Recently Dr. George Herschell[4] has advised the use of a rubber cord for the production of the resistance: he assumes that the evidence in favour of the practical utility of the resisted movements is overwhelming, and he points out the advantages which may be secured by the use of a mechanical apparatus. The exercising machine which he uses is called "The Whiteley Exerciser" (10 Ironmonger Lane, London, E.C.) By it we substitute for the fallible muscles of a nurse the unvarying tension of a rubber cord. It is equally astonishing that a saline bath at 94° for four minutes should in another case produce as striking a result; but either we must accept the results as facts, or else assume that numerous and accurate observers are capable of deceiving themselves and others by acts of jugglery with the sphygmograph and stethoscope.

On the **physiology of Nauheim treatment** many opinions were expressed. Dr. Harry Campbell's views are as follows:—The beneficial effect of cold baths and resisted movements is chiefly referable to two factors: a widespread tonic contraction of the muscles, and an increase in thoracic capacity. These two factors operate in both methods of treatment. Thus during the performance of a resisted movement in the upright position, almost the entire muscle-system is tonically contracted, in order to produce the necessary rigidity of the skeleton, and to preserve equilibrium; similarly, when the patient is put into a cold bath, an involuntary tonic contraction of the muscles occurs. That the mean respiratory capacity is increased during the performance of resisted movements is acknowledged by most authorities, and there can be no doubt that it is also increased by immersion in cold water. The advantage of this thoracic expansion is obvious, facilitating, as it does, the flow of blood through the lungs. The widespread tonic contraction has a threefold good effect: *a*, the

[1] *Lancet*, 1896, ii. 421. [2] *Brit. M. J.*, 1896, i. 1140. [3] *Lancet*, 1896, ii. 423. [4] *Ibid.*, 460.

heart is stimulated to more powerful contraction; *b*, blood is attracted into the muscles away from other areas; and *c*, the flow of blood into the right heart is retarded, the muscle and splanchnic veins being compressed by the contracted muscles. Dr. Watson Williams believes that the baths and exercises, carefully regulated, and with special directions as regards respiration, cause transudation from the capillaries into the lymphatics, relieve hydræmic plethora from which most of the cases are suffering, and by removing to a considerable extent the *vis a fronte* with which the heart has been unable to cope, thus placing it under favourable conditions for contracting with greater vigour and bringing the cardio-vascular system within the physiological range of Marey's law. Dr. Theodore Fisher commented on the kind of cases which derived most benefit from this kind of treatment, and he believed that it was in cases of hypertrophy without other organic defect in which he had seen best results. Probably it is not commonly successful in cases of valvular disease, but especially in the cases of alcoholic hypertrophy he had seen great improvement. In Sir T. Grainger Stewart's reply the further fact was elicited that as a result of his experiments he had come to the conclusion that it was the carbon dioxide in the water which was mainly instrumental in producing the marvellous results which had been seen.

Some other observers have suggested that the kind of massage due to the percussion on the body of innumerable bubbles of the carbonic acid gas is an important factor in the Nauheim treatment.

The method of **combined auscultation and percussion** is very closely associated with the Nauheim treatment, inasmuch as almost all the writers on the subject use the combined method for mapping out the heart-area as being more accurate and trustworthy than simple percussion. Those who have become accustomed to this combined method cannot fail to appreciate its advantages, not only as being useful in mapping out accurately the outlines of solid organs, but also for ascertaining the size and outlines of the hollow viscera. Within the last few years many communications have directed attention to the method. The most noteworthy are included in the list below.[1] There are many variations in the details adopted; the tuning-fork does not stand the test of experience; if worked by an electromagnet as suggested by Dr. A. L. Benedict, of Buffalo, it might be trustworthy, but who will care to carry about the needful apparatus for use at the bedside? But there is no need for any other apparatus than the ordinary binaural stethoscope held by

[1] F. W. Jackson, *N. York M. J.*, 1891, liii. 709; L. L. Seaman, *Tr. N. York Acad. M.*, 1891, 2nd s., vii. 279; Sir B. W. Richardson, *Lancet*, 1892, ii. 1037; C. E. Quimby, *Med. Rec.*, 1893, xliii. 353; W. Ewart, *Lancet*, 1893, i. 1241; A. H. Smith, *Med. Rec.*, 1893, xliii. 777; H. Sewall, *Med. News*, 1893, lxiii. 483; A. L. Benedict, *Tr. M. Soc. N. Y.*, 1895, 351; H. Royet, *Gaz. d. Hôp. de Toulouse*, 1896, x. 50; W. B. Thorne, *Brit. M. J.*, 1896, i. 653, 1140; Sir W. H. Broadbent, *Ibid.*, 769; G. Herschell, *Ibid.*, 943.

the one hand and the fingers of the other hand to be used as a direct percussor. The pencil is used by Dr. Bezly Thorne, but any kind of hammer will serve well for the purpose; that suggested by Dr. Kingscote, of Salisbury, and shown at the Carlisle meeting, should be efficient. This hammer is intended to be used with a boxwood cone, with a rubber ring surrounding the apex of the cone, and so limiting the extent of vibration through the surface-tissues. My usual habit has been to use the middle finger of the right hand flipped from the end of the thumb; but I take it that the method which is most familiar to the individual observer will be likely to give the best results. As regards the theory of the method, Dr. P. Watson Williams[1] says auscultatory percussion of the heart, which he has practised on the cadaver and verified the results by subsequent examination, while not an absolutely accurate method of determining the cardiac area in relation to the chest-wall, is nevertheless more accurate than any other method, and of sufficient accuracy for determining the cardiac area. But inasmuch as the curved surface of the heart is lying behind the curved surface of a thick chest-wall, the area of the chest's surface thus delineated and the actual area of the heart are in some measure comparable to the segments of a large and small circle having the same centre. Thus the cardiac area as mapped out on the surface of the chest must be larger than the actual cardiac area, and therefore any alteration in the cardiac area, however accurately determined, appears greater than actually occurs.

Intimately related to the Schotts' method comes the subject of **respiratory exercises in the treatment of disease**, on which a paper was read at Carlisle by Dr. Harry Campbell,[2] who stated that such exercises benefit not only by developing the lungs, and thus diminishing their liability to disease, but by facilitating the flow of blood and lymph. Dr. Campbell emphasised the importance of developing the lungs to the utmost in cases of heart-disease, for the better developed these organs the less is the work put upon the right heart; and since the limit of life in heart-disease is to a large extent determined by the length of time which the right heart can hold out, it is important to facilitate its work as far as possible. Special reference was made to the influence of diaphragmatic breathing. The movements of the diaphragm not only pump lymph from the peritoneum into the pleuræ, they also influence the functions of the abdominal and pelvic viscera by rhythmically compressing and (in many instances) dislocating these latter; hence diaphragmatic exercises are often useful in functional disturbances of these organs. Among the many diseases in which respiratory exercises are useful, emphysema was mentioned. It was pointed out that the chief trouble of the emphysematous patient is his inability to adequately expire, and that this is due to the fixation of the chest in a position of extreme inspiration. It was contended

[1] *Brit. M. J.*, 1896, ii. 714. [2] *Ibid.*, 716.

that this is due to the protracted over-action of the inspiratory muscles, no longer counteracted by normal pulmonary elasticity, and might be largely prevented by systematic, long-continued *ex*piratory exercises.

R. SHINGLETON SMITH.

### SURGERY.

